# The Paradox of Pulmonary Vascular Resistance: Restoration of Pulmonary Capillary Recruitment as a *Sine Qua Non* for True Therapeutic Success in Pulmonary Arterial Hypertension

**DOI:** 10.3390/jcm11154568

**Published:** 2022-08-05

**Authors:** David Langleben, Stylianos E. Orfanos, Benjamin D. Fox, Nathan Messas, Michele Giovinazzo, John D. Catravas

**Affiliations:** 1 Center for Pulmonary Vascular Disease, Azrieli Heart Center and Lady Davis Research Institute, Jewish General Hospital, McGill University, Montreal, QC H3T 1E2, Canada; 2 1st Department of Critical Care and Pulmonary Services, Pulmonary Hypertension Center, Evangelismos Hospital, National and Kapodistrian University of Athens Medical School, 10676 Athens, Greece; 3 Pulmonary Division, Yitzchak Shamir Hospital, Tel Aviv University, Tzrifin 69978, Israel; 4 Frank Reidy Research Center for Bioelectrics, Old Dominion University, Norfolk, VA 23529, USA

**Keywords:** pulmonary arterial hypertension, hemodynamics, exercise, pulmonary hypertension therapy, pulmonary microvasculature, pulmonary capillary, pulmonary vascular resistance

## Abstract

Exercise-induced increases in pulmonary blood flow normally increase pulmonary arterial pressure only minimally, largely due to a reserve of pulmonary capillaries that are available for recruitment to carry the flow. In pulmonary arterial hypertension, due to precapillary arteriolar obstruction, such recruitment is greatly reduced. In exercising pulmonary arterial hypertension patients, pulmonary arterial pressure remains high and may even increase further. Current pulmonary arterial hypertension therapies, acting principally as vasodilators, decrease calculated pulmonary vascular resistance by increasing pulmonary blood flow but have a minimal effect in lowering pulmonary arterial pressure and do not restore significant capillary recruitment. Novel pulmonary arterial hypertension therapies that have mainly antiproliferative properties are being developed to try and diminish proliferative cellular obstruction in precapillary arterioles. If effective, those agents should restore capillary recruitment and, during exercise testing, pulmonary arterial pressure should remain low despite increasing pulmonary blood flow. The effectiveness of every novel therapy for pulmonary arterial hypertension should be evaluated not only at rest, but with measurement of exercise pulmonary hemodynamics during clinical trials.

Normal human pulmonary circulation has the remarkable ability to accept major increases in blood flow with only minor rises in pulmonary artery pressure, best exemplified by the hemodynamic response to exercise [1,2,3,4]. The lungs have an extremely large capillary surface area, equivalent to approximately two tennis courts in an adult. However, at rest, only a fraction of that surface area is being perfused, with a large number of non-concomitantly perfused capillaries available for low driving pressure-recruitment as the demand for accommodation of cardiac output increases [5,6,7,8]. As an example, one need only observe water finding an expanding surface area on a tabletop to understand that a high driving pressure is not required to optimize the surface area utilized. As blood flow continues to rise, maximal regional recruitment is attained, and any further increases in flow result in distention of already perfused capillaries.

The development of techniques to measure the first-pass transpulmonary metabolism of radio-labelled peptides has enabled the identification and analysis of the recruitment and distention phases of capillary blood flow [9]. The pulmonary endothelial luminal surface is the major site of angiotensin-1 ectoenzyme (ACE-1) activity, and the degree of first-pass ACE-1 substrate transformation is proportional to the amount of perfused functional capillary surface area. Trace quantities of a radiolabelled, hemodynamically inactive tripeptide (^3^H-benzoyl-Phe-Ala-Pro, BPAP) are injected into the lung, and the first-pass transpulmonary metabolism can be measured by collecting systemic arterial blood that contains the pulmonary venous effluent. The instantaneous hydrolysis and percent metabolism of BPAP are determined and the functional capillary surface area is calculated. The technique has been validated in several species of animals, where the transition from capillary recruitment to capillary distention was shown [10,11]. Its utility was subsequently demonstrated in humans, where single- and double-lung injections of BPAP established the range of normal values for functional capillary surface area [12]. Reduced functional capillary surface area has been described in acute lung injury, early scleroderma, and in some types of pulmonary hypertension [13,14,15]. Of great relevance to the current discussion, the recruitment/distention curve has been studied in normal exercising humans and a comparison with other mammalian species shows similar ranges of blood flow during the transition from predominant recruitment to predominant distention [16,17]. During the recruitment phase, as opposed to complete distention, further functional capillary surface area is exposed as flow increases (Figure 1).

Pulmonary arterial hypertension is a devastating group of disorders in which progressive structural remodeling and luminal obliteration of upstream pulmonary arterioles cause a heterogenous reduction in perfused downstream capillary surface area [18,19]. By the time patients present at a pulmonary hypertension clinic with World Health Organization functional class III dyspnea, their perfused capillary surface area at rest may be 30% of normal [14]. The progressive rise in pulmonary arterial pressure causes right heart failure, reduced cardiac output and ultimately death [20]. The heterogeneous histologic abnormalities result in acinar capillaries that are fed by less affected arterioles carrying normal or supernormal flow to support the cardiac output, while flow is extremely reduced or absent in capillaries downstream from more affected arterioles [21]. Although cardiac output and thus pulmonary blood flow in these sick patients may be extremely low, it is possible that even that decreased blood flow results in complete capillary recruitment, and even distention, in the reduced number of perfused capillaries available to carry it (Figure 1).

The presently approved mainstays of pulmonary arterial hypertension therapy involve rebalancing abnormalities in the prostacyclin, nitric oxide and endothelin-1 pathways [22]. These “vasodilator” treatments have provided significant benefit to patients, but are not curative, and there is no evidence of significant impact on the arteriolar obstruction in humans even after years of therapy [23]. Although the medications lower calculated pulmonary vascular resistance mainly via increased cardiac output, some patients may have a larger decrease in pulmonary arterial pressure [24] and most patients have only a small improvement, even with prolonged therapy [25,26]. An increased cardiac output can only be beneficial in a pulmonary arterial hypertension patient, and a decrease in calculated resting pulmonary vascular resistance has been used as the primary endpoint in the phase 2 studies that contributed to the clinical approval of the majority of our current pulmonary arterial hypertension therapies. However, in most patients, the question remains: does that reduction in resting pulmonary vascular resistance represent a gain in perfused capillary surface area, which would indicate a true restoration of lung circulatory function [27]? The analysis below suggests not.

Insights can be gained from observations during acute vasodilator studies in patients with idiopathic pulmonary arterial hypertension that have identified two types of hemodynamic response [28]. These two different responses likely represent two different pathologies, and studies suggest a different genetic basis [29,30,31]. The more common “resistance” response is as described above (i.e., increased resting cardiac output with minimal or no reduction in pulmonary artery pressure, and decreased resting pulmonary vascular resistance). In a second group, termed “pressure” responders, resting pulmonary vascular resistance falls, but pulmonary artery pressure falls importantly even as resting cardiac output increases. However, although decreased resting pulmonary vascular resistance does help patients clinically, it alone is unable to distinguish between the two groups [27].

Moreover, the responses of the two aforementioned groups to the vasodilator, in terms of change in capillary surface area, are completely different. When we explore relationships in the raw data of a previous vasodilator study, the differences between the two groups are apparent [32]. The “resistance” responders only increased capillary surface area by a mean of 7% [32]. When the results are viewed in terms of the final functional capillary surface area attained, versus the final hemodynamic levels attained, it is evident that none of the “resistance” responders achieved functional capillary surface area levels anywhere approaching those of the “pressure” responders, who attained the expected normal levels of functional capillary surface area (Figure 2).

In the “resistance” response group, there was a wide range of pulmonary artery pressures and cardiac outputs which had no impact on the final functional capillary surface area. Furthermore, the absolute level of pulmonary vascular resistance had no relationship to the functional capillary surface area. These data do not support the hypothesis that the level of pulmonary vascular resistance is in-itself indicative of the degree of microvascular perfusion. However, one of the standard criteria for judging the success of therapy is the degree of reduction in pulmonary vascular resistance. All but one of the “resistance” responders had a decrease in pulmonary vascular resistance, with some quite impressively reaching the degree seen in “pressure” responders (Figure 3). However, there was no relationship between change in pulmonary vascular resistance and functional capillary surface area, which remained very low. In those patients, it is likely that the vasodilator acted only on unaffected or minimally diseased arterioles that were already carrying flow to their downstream capillaries, and they were not able to recruit additional capillary surface area.

By contrast, the very rare “pressure” responders have a vascular phenotype that involves principally vasoconstriction rather than cellular obstruction, and they increase functional capillary surface area by 46% with the vasodilator [32]. They achieve a high-normal cardiac output, and only slightly elevated pulmonary vascular pressures and pulmonary vascular resistances (Figure 2). They also have a large reduction in pulmonary vascular resistance (Figure 3), but it should be noted that is determined by a large decrease in pulmonary artery pressure with the increased cardiac output.

Although based on the data from only one study, the implications of the above analysis are that the “resistance” responders, the great majority of pulmonary arterial hypertension patients, have a markedly reduced perfused capillary surface area due to upstream arteriolar obstruction, that their remaining perfused capillary surface area is maximally recruited, and that any increased blood flow with “vasodilator” therapy is handled by capillary distention of the available vascular bed rather than recruitment (Figure 4). Despite certain clinical benefits, this pulmonary vascular resistance response to therapy does not represent a return to a normal pattern of pulmonary vascular function. By contrast, the “pressure” response offers a glimpse and a paradigm of the effect that successful “antiproliferative” therapies could have on recruitable capillary surface area in the “resistance” group that suffers from cellular arteriolar obstruction (Figure 4).

Exercise testing with concurrent hemodynamic measurement has provided insights into pulmonary circulatory and cardiac dysfunction in pulmonary hypertension [33,34,35,36,37,38]. A variety of ventilatory parameters, including ventilatory dead space indicative of ventilation/perfusion matching, are abnormal, and the pulmonary artery pressure/blood flow slope is abnormally steep. The capillary surface area response to exercise is also abnormal in pulmonary arterial hypertension (Figure 4). In a small number of such patients who performed symptom-limited exercise, we have not seen an increase in functional capillary surface area, showing a lack of recruitment. This has also been detected indirectly by others, in that patients with pulmonary arterial hypertension have an abnormally high ventilatory dead space [35].

With the realization that abnormal vascular cell proliferation is central to the arteriolar narrowing in pulmonary arterial hypertension, novel therapeutic agents that affect cell growth rather than vascular tone are being studied [39]. Based on the above, a truly effective therapy should result in more perfused capillary surface area at rest but should also restore capillary recruitment on exercise. The hemodynamic appearance at rest after antiproliferative therapy might show a decreased pulmonary artery pressure and increased cardiac output. However, it is equally possible that resting pulmonary artery pressure might fall without much or any further increase in already normalized cardiac output in patients already receiving background vasodilator pulmonary arterial hypertension therapy. Both patterns of response would indicate less arteriolar obstruction and greater availability of downstream capillary surface area for recruitment. Just as water finds a greater area if constraints to spreading on a surface are removed, an unchanged pulmonary blood flow can distribute itself more widely if more surface area can be reached for perfusion. A recent phase 2 study with the TGF-β superfamily ligand trap, sotatercept, found a decreased pulmonary artery pressure but minimal change in resting flow, consistent with the latter of the above phenomena, and other potentially antiproliferative agents are also under evaluation in clinical trials described elsewhere [40,41]. By contrast, the antiproliferative tyrosine kinase inhibitor, imatinib, increased cardiac output with a smaller but significant decrease in pulmonary arterial pressure [42]. Thus, both hemodynamic patterns have been observed with antiproliferative therapy. An even more impressive indicator of success would be an improvement in exercise hemodynamics after therapy, with minimal rise in pulmonary artery pressure despite a large rise in cardiac output. That would indicate restoration of capillary surface area recruitment with flow. At least one such exercise study is underway (SPECTRA, NCT03738150). It is unlikely that any future effective PAH therapy will improve exercise hemodynamics without first demonstrating a marked improvement in resting mean pulmonary artery pressure, as is discussed above. The threshold for that resting pressure is unknown, but is likely 30 mm Hg or less with a normal cardiac output. Future studies should search for this threshold and correlate it to normalization of the exercise response.

**Conclusions:** Improvement in pulmonary vascular resistance has been an important endpoint in many clinical studies, and its value has been proven in terms of clinical outcomes and prognosis [43]. However, the use of a reduction in pulmonary vascular resistance as an indicator of therapeutic success, when it is driven mainly by increased cardiac output rather than by large decreases in pulmonary arterial pressure, may be misleading with regard to a medication’s ability to reverse cellular pulmonary microvascular obstruction and to restore capillary recruitment. The metabolic analysis of pulmonary capillary surface area is an extremely powerful research tool to aid in the understanding of physiological microcirculatory function in health and disease [12]. However, it is technically complex and involves the injection of a radiolabelled peptide, and will therefore be difficult to use broadly as a clinical tool. Exercise hemodynamics can provide surrogate information, and invasive cardiopulmonary exercise testing can be used widely, as long as it is performed and interpreted appropriately [33]. Certainly, to understand the effects of future pulmonary arterial hypertension therapies, measurement of exercise hemodynamics, as a stand-alone trial or a sub-study performed at expert centers, should form part of the evaluation of any novel treatment. Re-establishment of the normal reserve of recruitable capillary surface area in patients should be the objective of every pulmonary arterial hypertension therapy.

## Figures and Tables

**Figure 1:**
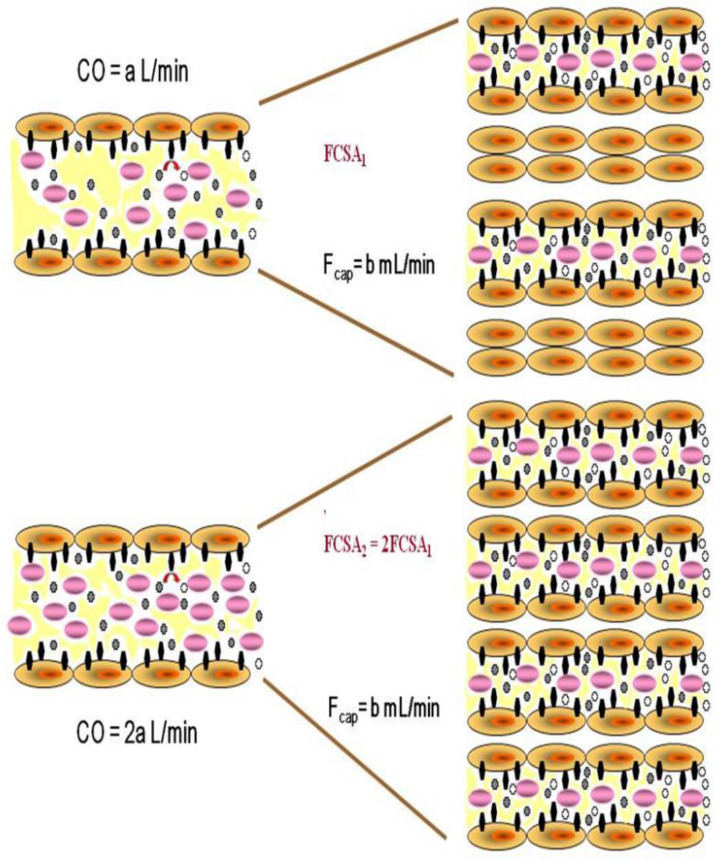
Schematic representation of increased functional capillary surface area (FCSA) during capillary recruitment, as cardiac output (CO) and thus pulmonary blood flow doubles. Individual capillary flow (Fcap) remains unchanged as more capillaries are recruited to accept the total blood flow. However, the surface area and the number of ectoenzyme sites available for substrate metabolism increases. Black symbols represent enzymatic sites, purple symbols represent blood cells, small black ovals represent unmetabolized substrate and small ovals represent metabolized substrate.

**Figure 2:**
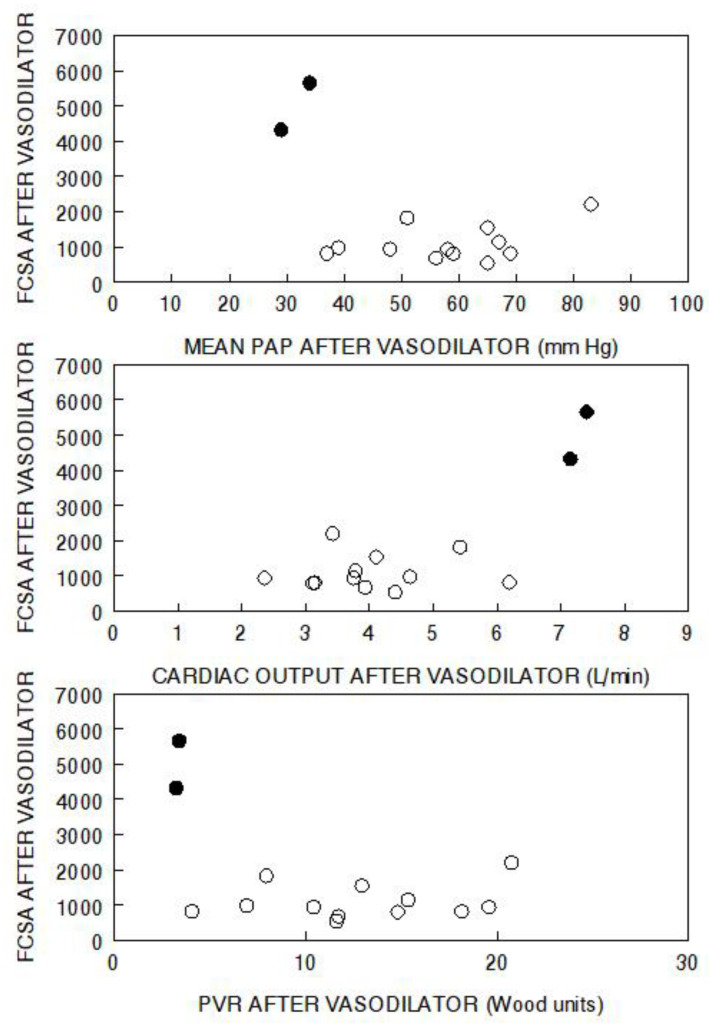
Functional capillary surface area normalized to body surface area (FCSA, mL/min/m^2^) in human lungs post acute vasodilator therapy, as compared to pulmonary artery pressure (PAP), cardiac output, and pulmonary vascular resistance (PVR) post vasodilator. The open circles represent “resistance” responders and the filled circles represent “pressure” responders. Data have been adapted from reference [32].

**Figure 3:**
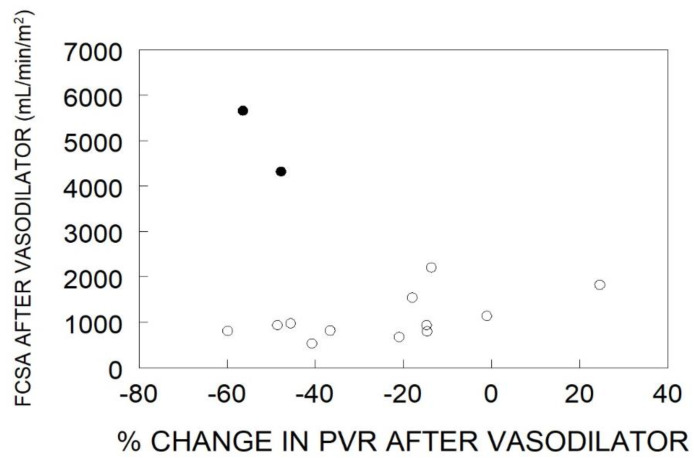
Functional capillary surface area normalized to body surface area (FCSA, mL/min/m^2^) in human lungs post acute vasodilator therapy, as compared to percent change in pulmonary vascular resistance (PVR) after administration of a vasodilator. The open circles represent “resistance” responders and the filled circles represent “pressure” responders. Data have been adapted from reference [32].

**Figure 4:**
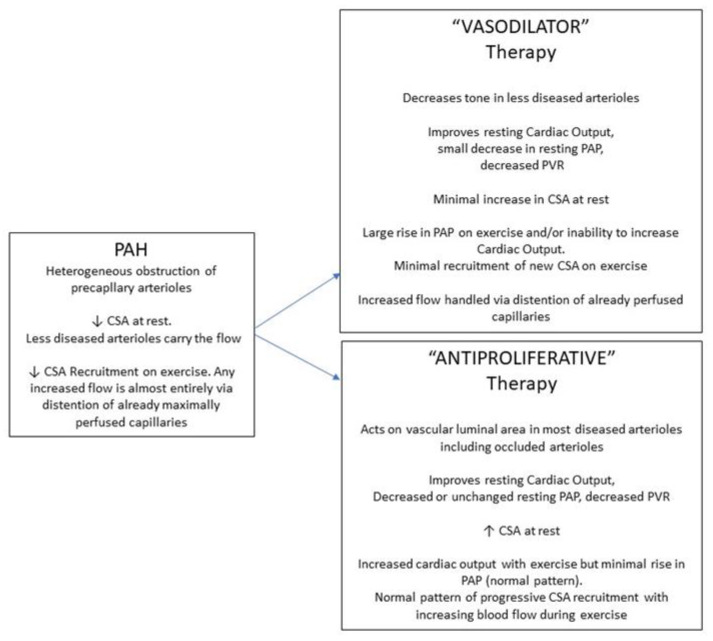
Schematic representation of the effects of pulmonary arterial hypertension (PAH) on pulmonary capillary surface area (CSA). Shown are the potential effect that vasodilator-type therapies (VASODILATOR), and therapies that would affect cellular proliferative obstruction (ANTIPROLIFERATIVE), would have on hemodynamics (PAP, pulmonary artery pressure; PVR, pulmonary vascular resistance) and CSA, at rest and upon exercise.

## Data Availability

Not applicable.
